# Pharmacodynamic Material Basis and Potential Mechanism Study of Spatholobi Caulis in Reversing Osteoporosis

**DOI:** 10.1155/2023/3071147

**Published:** 2023-04-14

**Authors:** Jianpeng Xiao, Wei Shang, Zhiming Zhao, Jun Jiang, Jianping Chen, Hui Cai, Jinjin He, Zhihui Cai, Zihan Zhao

**Affiliations:** ^1^Department of TCM, Jinling Hospital, School of Medicine, Nanjing University, Nanjing, China; ^2^School of Pharmacy, Jiangsu University, 301^#^ Xuefu Road, Zhenjiang 212013, Jiangsu, China; ^3^School of Chinese Medicine, The University of Hong Kong, 10 Sassoon Road, Pokfulam, Hong Kong, China

## Abstract

**Objective:**

To elucidate the mechanism of Spatholobi Caulis (SC) in treating osteoporosis (OP) integrated zebrafish model and bioinformatics.

**Methods:**

Skeleton staining coupled with image quantification was performed to evaluate the effects of SC on skeleton mineralization area (SSA) and total optical density (TOD). Zebrafish locomotor activity was monitored using the EthoVision XT. Bioactive compounds of SC and their corresponding protein targets were acquired from Traditional Chinese Medicine Systems Pharmacology (TCMSP) database. Potential therapeutic targets for OP were summarized through retrieving 5 databases, and then, the overlapping genes between SC and OP were acquired. The core genes were selected by CytoHubba. Subsequently, Kyoto Encyclopedia of Genes and Genomes (KEGG) pathway and Gene Ontology (GO) functional analysis of the intersection target genes were carried out by R software. Finally, the molecular docking simulation was manipulated between the ingredients and the hub genes.

**Results:**

Compared with the model group, SC significantly increased the SSA and TOD at 10 mg/mL and improved the locomotor activity in a dose-dependent manner (*p* < 0.001). 33 components of SC were associated with 72 OP-related genes including 10 core genes (MAPK1, VEGFA, MMP9, AKT1, AR, IL6, CALM3, TP53, EGFR, and CAT). Advanced Glycation End Product (AGE) Receptor for AGE (RAGE) signaling pathway was screened out as the principal pathway of SC in anti-OP. The bioactive components (Aloe-emodin, Emodin, Formononetin, Licochalcone A, Luteolin, and Lopac-I-3766) have excellent affinity to core genes (MAPK1, VEGFA, MMP9, AKT1, and IL6).

**Conclusion:**

SC had the hierarchical network characteristics of “multicomponents/multitargets/multifunctions/multipathways” in reversing OP, but AGE-RAGE signaling pathway may be the main regulatory mechanism.

## 1. Introduction

Osteoporosis (OP) is a common systemic bone disease with a significant impact on human health and characterized by low bone quality, low bone density, and bone microstructure destruction, which easily leads to decreased bone strength and increased fracture risk [1–3]. OP has been considered a silent disease, for it is usually asymptomatic and cannot be detected until the first fracture occurs [4–6]. According to statistics released by international osteoporosis foundation, OP worldwide causes more than 8.9 million fractures annually, and this number is projected to be tripled by 2050 [7, 8]. The increasing incidence of OP places a heavy social and economic burden on the health care system [9–11]. Despite the fact that substantial pharmacological options have been approved for the management of OP, the potential long-term adverse effects of anti-OP medications and poor adherence to the treatment urgently await more effective strategies to satisfy the unmet medical needs [12–15]. Alternative natural medicine may play an emerging role in against OP by complementarily overcoming the limitations of the current marketed medications [16–18].

Spatholobi Caulis (SC), known as Jixueteng in Chinese Pharmacopoeia (2020 version), is the vine stem of *Spatholobus suberectus* Dunn of the family Leguminosae [19]. SC is a Chinese medicine that can promote blood circulation, dispel stasis, inhibit platelet aggregation, stimulate hematopoiesis, and treat anemia and diseases related to blood stasis syndrome [19–21]. In consequence, SC has been mainly prescribed for the treatment of irregular menstruation [22, 23], dysmenorrhea [24], amenorrhea [25], rheumatism [26], blood deficiency, and chlorosis [27]. Increasing evidence suggested that SC had massive pharmacological properties, including antioxidation [28], anti-infection [29], anticancer [30], and promoted angiogenesis [31]. In addition, it was reported that SC exerted anti-OP through inhibition of osteoclastogenesis and stimulation of chondrogenesis [32]. Also, recent paper demonstrated that AGE-RAGE pathway plays a vital part in OP treatment after intervention by SC [33, 34]. However, studies on the mechanisms underlying SC's antiosteoporotic effects are still lacking.

As a new interdisciplinary developed in recent years, network pharmacology provides a “multiway,” “multitarget” method for drug analysis and is similar with the thoughts of traditional Chinese medicine (TCM) compound prescription, which concurs with the characteristics of the overall compatibility of TCM and comprehensive and multipathway treatment of diseases [35]. Recently, numerous studies illustrated that network pharmacology can reach high performance on prediction the mechanism of Chinese medicine, indicating that it might become a powerful tool to systematically explore the new application of TCM [36, 37]. In this study, prednisolone- (PNSL-) induced zebrafish OP model was employed to assess the therapeutic effect of SC. Subsequently, we adopted network pharmacology to explore the underlying mechanism of SC's antiosteoporotic effects. The major bioactive components of SC and corresponding targets were obtained, and “drug-active components-diseasetargets-signaling pathways” network was constructed. Ultimately, the potential mechanism of SC in the treatment of OP was revealed via a combination of Gene Ontology (GO) function analysis and Kyoto Encyclopedia of Genes and Genomes (KEGG) pathway enrichment analysis.

## 2. Materials and Methods

### 2.1. Experimental Animal

Zebrafish larvae were purchased from Nanjing Yishu Lihua Biotechnology Co., Ltd and cultured in blank E3 medium (containing 0.33 mM CaCl_2_, 0.33 mM MgSO_4,_ 5 mM NaCl, and 0.17 mM KCl). Zebrafish embryos were cultured in Intelligent light incubator with a 14/10 h light/dark cycle [38, 39]. Animal experiments were carried out in accordance with the Guidelines for Animal Experimentation of Jiangsu University (Zhenjiang, China), and the protocol was approved by the Animal Ethics Committee of this institution.

### 2.2. Experimental Design

Newly hatched zebrafish larvae at the age of 3 days after fertilization (DAF) were placed into 6-well plates (15 larvae in each well) [40, 41]. The zebrafish larvae were randomly divided into 7 groups as follows: Control (CON, blank medium), dimethyl sulfoxide (DMSO, 0.5% DMSO), Model group (MX, 25 *μ*M PNSL), Etidronate Disodium group (ED, 15 mM ED + 25 *μ*M PNSL), SC-H (10 mg/mL SC + 25 *μ*M PNSL), SC-M (1 mg/mL SC + 25 *μ*M PNSL), and SC-L (0.1 mg/mL SC + 25 *μ*M PNSL).

### 2.3. Observation of Zebrafish Locomotor Activity

After 4-day drug deliveries, zebrafish locomotor activity was monitored by EthoVision behavior system [42, 43]. The instrument parameters are set as follows: “prior to the start of tracking, the software needed to be calibrated; the video sampling rate was 25 frames per second (fps), based on the design recommendations [44]; first, under the Trial List, one trial was selected. For the Arena Settings, each well/arena was calibrated based on the diameter of the well [45]. The diameter of the wells within the well plates used in this manuscript (96-well plate) is 6.54 mm [46]. For the Detection Settings, dynamic subtraction was selected, and the dark contrast and subject contour were adjusted to optimize tracking efficiency [47, 48]. Within the Analysis Profiles, the selected dependent variables were distance moved, velocity, and time spent moving [49]. These endpoints were based on the larvae's center-point activity [50]. The results were then exported to Excel and statistical analysis software suites.” According to the methods described above, moving distance (MD), moving speed (AS), travel frequency (TF), and hotspot are selected as the anti-OP drug efficiency evaluation indexes in this model.

### 2.4. Alizarin Red Staining

At the 10 DAF, all zebrafish larvae were collected and killed under anesthesia by 3-Aminobenzoic acid ethyl ester methanesulfonate (MS-222, 100 mg/L) [51]. After removing MS-222 solution, 4% paraformaldehyde was utilized to fix zebrafish and stained with 0.01% Alizarin Red Staining (ARS, containing 0.5% KOH) overnight [52]. After removal of ARS solution, newly prepared bleaching solution containing 1.5% H_2_O_2_ and 0.5% KOH was added [53]. 1.5 hours later, all zebrafish had been decolorized and preserved in different ratios of glycerol and 0.5% KOH solution [54]. The stained bones of zebrafish were observed under a microscope (Olympus IX71/IX81, Olympus Corporation, Japan). Digital images [55] were analyzed for quantification of skeleton stained area using ImageJ software. Three replicates were performed.

### 2.5. Screening of Active Compounds and Corresponding Target Genes in SC

Traditional Chinese Medicine Systems Pharmacology database (TCMSP) was utilized to search the bioactive components of SC, with the screening conditions of “oral bioavailability (OB) ≥20% and drug like (DL) ≥0.1.” Meanwhile, the corresponding target genes of the above components were obtained [56, 57].

### 2.6. Mining of OP-Related Targets

OP-related targets were collected through retrieving GeneCards [58] (https://www.genecards.org/), Online Mendelian Inheritance in Man (OMIM) [59] (https://omim.org/, updated November 25, 2020), PharmGkb [60] (https://www.pharmgkb.org/), Therapeutic Target Database (TTD) [61] (https://db.idrblab.net/ttd/), and DrugBank [62] (https://www.drugbank.org/) using the keyword “osteoporosis.” The OP-related target genes were gathered, and Venn diagram was constructed.

### 2.7. Overlapping Targets of Drug Potential Genes and OP-Related Genes

R language [63] was applied to obtain the overlapping targets of drug potential genes and OP-related genes. These common target genes were used for further analysis.

### 2.8. Protein-Protein Interaction (PPI) Network and Hub Genes

PPI network was constructed when the shared target genes were imported into the STRING database [64]. Subsequently, 12 algorithms in CytoHubba, that is, Betweenness, BottleNeck, Closeness, ClusteringCoefficient, Degree, Density of Maximum Neighborhood Component (DMNC), EcCentricity, Edge Percolated Component (EPC), Maximal Clique Centrality (MCC), Maximum Neighborhood Component (MNC), Radiality, and Stress, were utilized to screen the top 10 core target genes [65, 66].

### 2.9. GO and KEGG Analysis

R language and Bioconductor platform were used to perform GO enrichment analysis and KEGG pathway analysis. The first 10 functional categories of biological process (BP), cellular component (CC), and molecular function (MF) were screened out to construct the histogram and bubble diagram. Then, the target proteins were analyzed using the KEGG enrichment analysis, and the top 30 signaling pathways were chosen to draw the histogram and bubble diagram.

### 2.10. Network Visualization of “Active Ingredients-Potential Targets-Signaling Pathways”

Cytoscape software was adopted to draw a ternary network including bioactive ingredients-target genes-signaling pathways. The above bioactive components and corresponding genes were imported into the software, and the ternary network “drug-active components-potential therapeutic targets of disease-relevant signaling pathways” was drawn [67, 68].

### 2.11. Molecular Docking Analysis

The plug-in CytoHubba of Cytoscape software was used to screen top 10 hub genes. The core genes were selected to find the related drug components in the compound regulatory network as small molecular ligands. Molecular docking simulation was performed as previously described. The specific process is as follows: “the 2D structure information of drug chemical components was downloaded from PubChem (https://www.ncbi.nlm.nih.gov/) platform and converted into the 3D structure by ChemBio3D software, and the energy optimization of MM2 was carried out to complete the preparation of small molecule ligands. The 3D structure of the candidate target proteins was downloaded from PDB (https://www.rcsb.org/) database, and then, the protein receptors were prepared after the water molecules, and ligands were removed by PyMOL2.4.0 software. AutoDockTools software was used to read the receptor files, which were converted to PDBQT format after hydrotreating ion modification. The ligand files were also converted to PDBQT format for saving and then converted into the 2D structure to draw the active pockets. Finally, AutoDock vina software will be used for molecular docking, and the lowest free energy model is selected for visual analysis.”

### 2.12. Statistical Analysis

The GraphPad Prism 5 software [69–71]was used for statistical analysis, and the data was expressed as the mean ± SD. The differences were performed by one-way analysis of variance, and *p* < 0.05 was considered significant difference [39, 72, 73].

## 3. Results

### 3.1. Protective Effect of SC on the Zebrafish OP Model Induced by PNSL

**Figure 1:**
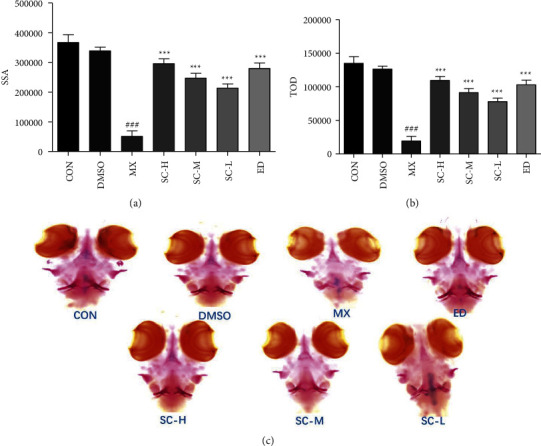
The effect of SC on mineralization in zebrafish larvae (*n* = 15). (a) Skeleton stained area (SSA). (b) Total optical density (TOD). (c) Ventral view of alizarin red stained zebrafish skull (×100). CON, blank *E*_3_ medium; DMSO, 0.5% DMSO; MX, 25 *μ*M PNSL; ED, 15 *μ*M ED + 25 *μ*M PNSL; SC-H, 10.0 *μ*g/mL SC + 25 *μ*M PNSL; SC-M, 1.0 *μ*g/mL SC + 25 *μ*M PNSL; SC-L, 0.1 *μ*g/mL SC + 25 *μ*M PNSL. ^###^*p* < 0.001 compared with DMSO. ^*∗∗∗*^*p* < 0.001 compared with MX.

At the 3DAF, zebrafish were incubated with PNSL for three days in 6 well plates to establish a rapid OP model. The SSA and TOD, which represented osteoblast differentiation, were used to determine the amount of bone mineralization of zebrafish larvae. SSA and TOD of the zebrafish larvae were significantly lower in the PNSL-treated groups than in the DMSO with values of 51423.33 and 19405.03, respectively, indicating that PNSL reduced bone mineralization and inhibited osteogenic differentiation in zebrafish larvae (Figure 1). When PNSL-induced zebrafish was treated with 0.1, 1, and 10 mg/mL SC, higher mineralization of the vertebrate column was discovered in a dose-dependent pattern. SSA and TOD were significantly increased to 295764.70 and 109542.50 at 10 mg/mL, respectively, as shown in Figure 1. In conclusion, the study indicated that SC could reverse the bone loss of zebrafish induced by PNSL.

### 3.2. Protective Effect of SC on Zebrafish Locomotor Activity

**Figure 2:**
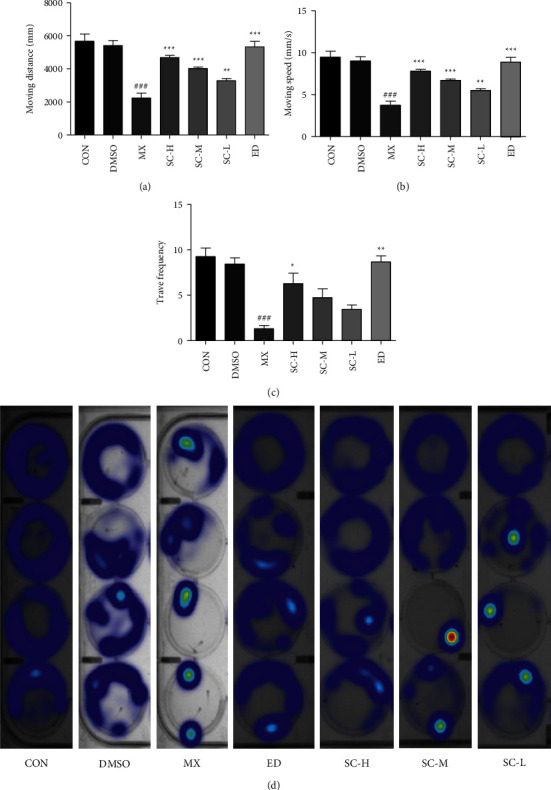
Effect of SC on zebrafish locomotor activity (*n* = 15). (a) Moving distance (MD); (b) moving speed (MS); (c) travel frequency (TF); (d) hot plot. CON, blank *E*_3_ medium; DMSO, 0.5% DMSO; MX, 25 *μ*M PNSL; ED, 15 *μ*M ED + 25 *μ*M PNSL; SC-H, 10.0 *μ*g/mL SC + 25 *μ*M PNSL; SC-M, 1.0 *μ*g/mL SC + 25 *μ*M PNSL; SC-L, 0.1 *μ*g/mL SC + 25 *μ*M PNSL. ^###^*p* < 0.001 compared with DMSO. ^*∗*^*p* < 0.05 compared with MX. ^*∗∗*^*p* < 0.01 compared with MX. ^*∗∗∗*^*p* < 0.001 compared with MX.

The behavior analyzer EthoVision was used to track the movement of zebrafish. As shown in Figures 2(a)–2(c), the Moving speed (MS), moving distance (MD), and travel frequency (TF) of PNSL group were significantly lower than those of the CON, while for SC treatment group, these parameters were close to the DMSO. The results showed that different concentrations of SC increased MD by 109.46%, 79.91%, and 47.27%, respectively, with partial significance (*p* < 0.001 − 0.01). TF for SC-H, SC-M, and SC-L was 371.43%, 254.54%, and 159.62%, respectively. Also, the hot plot revealed zebrafish activity degree (Figure 2(d)). These results with significant differences demonstrated that SC can reverse OP.

### 3.3. Main Components of SC and Treatment Targets of OP

68 active components in SC were obtained through retrieving the TCMSP database, and after screening by “OB ≥20% and DL ≥0.1,” 33 chemical bioactive components of SC were obtained (Table 1). 609 corresponding genes were collected by UniProt gene annotation simultaneously.

**Figure 3:**
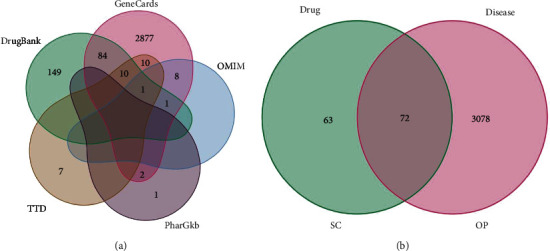
Venn diagram of OP-related disease targets and overlapping targets between OP-related and SC-related targets. (a) OP-related disease targets; (b) overlapping targets between OP-related and SC-related targets.

Through PharmGkb, TTD, DrugBank, GeneCards, and OMIM, 3150 OP-related targets were collected (Figure 3(a)). By matching the target of SC with OP-related targets, 72 common genes were derived, which were potential targets of SC in treating OP (Table 2, Figure 3(b)).

### 3.4. Construction of PPI Network and Acquisition of Core Genes

**Figure 4:**
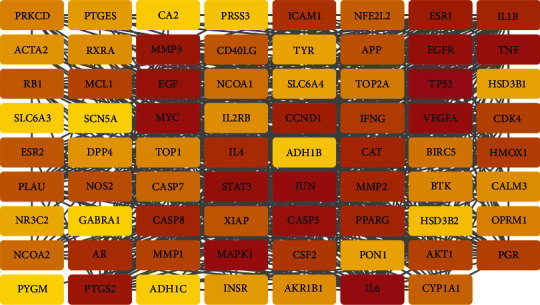
Protein-protein interaction network (PPI). The PPI network from STRING was further analyzed using Cytoscape software (the line between two nodes indicates the interaction; the darker the color of the node, the more the relationship between them).

**Figure 5:**
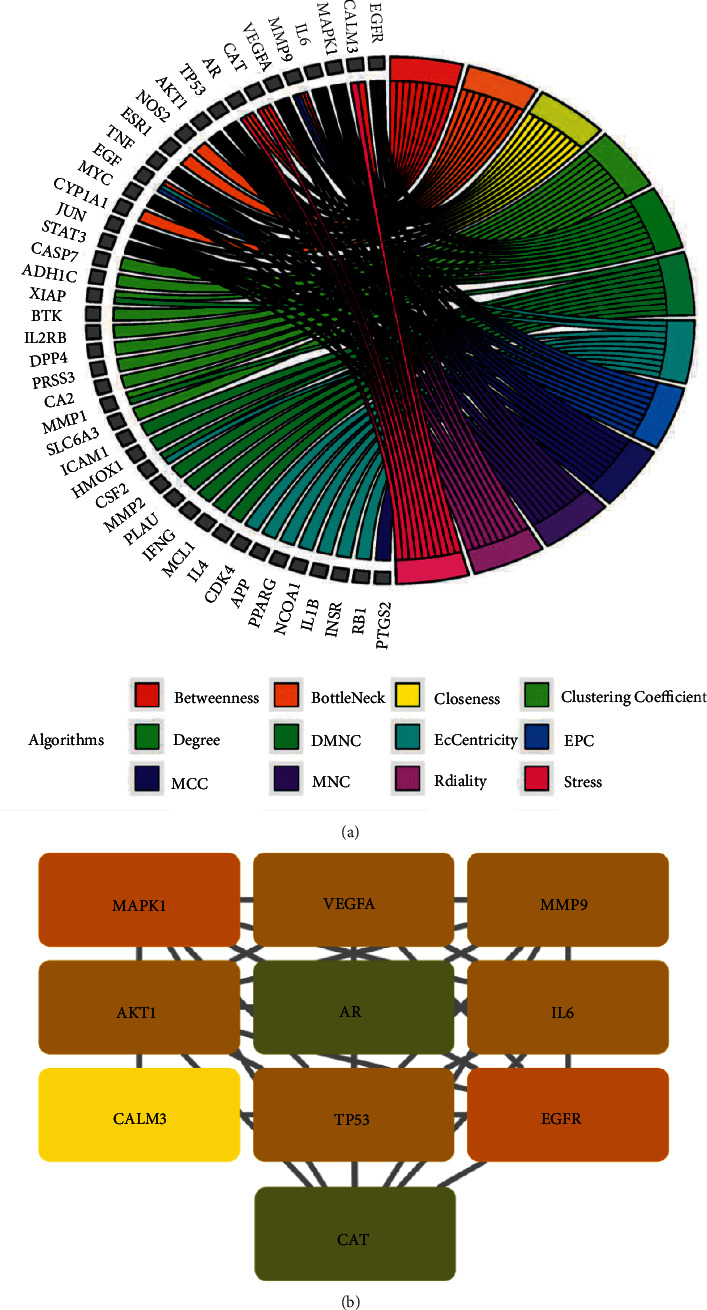
Analysis of 10 main core target genes. (a) Chord diagram of the corresponding relationship between the top 10 genes and 12 cytohubba algorithms. (b) The top 10 hub genes.

The 72 obtained intersecting genes were imported into the STRING database to obtain the interaction relationships between them and save the data as a file in TSV format (Figure 4). Secondly, we visualized the PPI network by importing it into the Cytoscape software and identified 10 core genes using plug in-CytoHubba of Cytoscape software, namely, MAPK1, VEGFA, MMP9, AKT1, AR, IL6, CALM3, TP53, EGFR, and CAT (Figure 5).

### 3.5. GO and KEGG Enrichment Analysis

**Figure 6:**
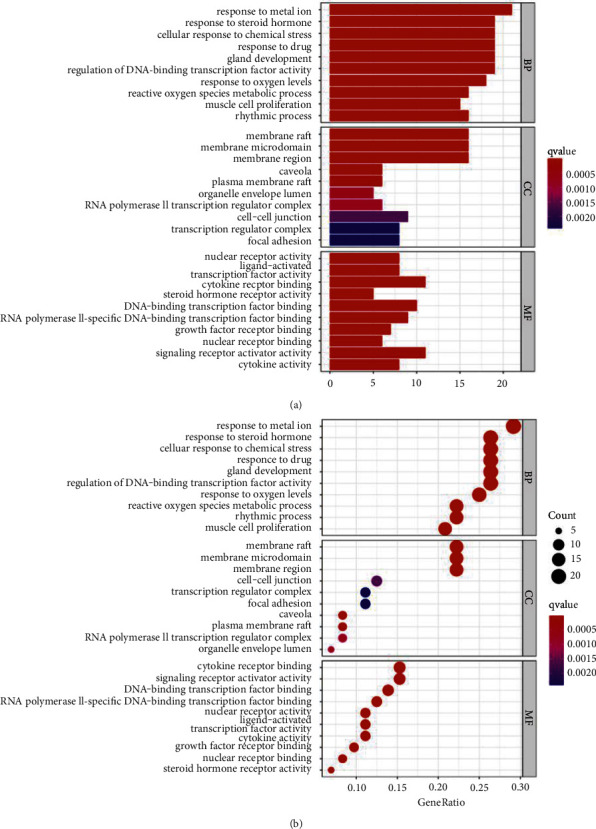
GO function analysis. (a) Barplot; (b) barble, including BP, MF, and CC. The *y*-axis shows top 10 significantly enriched BP, CC, and MF categories, and the *x*-axis displays the number of enrichment genes of these terms (*p* < 0.05). The color of each bar represents the adjusted *p* value, and the length represents the number of enriched genes.

In order to further elucidate the potential relationship between common genes and the mechanism by which the SC might remedy OP, R language was applied to perform GO and KEGG analysis. A total of 2049 GO terms were collected, of which 1892 are biological process (BP) entries, 106 molecular function (MF) entries, and 51 cellular component (CC) entries. As shown in Figure 6, the top 10 BP terms were principally relevant to the response to steroid hormone, response to metal ion, cellular response to chemical stress, response to drug, gland development, regulation of DNA-binding transcription factor activity, response to oxygen levels, reactive oxygen species metabolic process, muscle cell proliferation, and rhythmic process. The MF analysis indicated that these gene targets were mainly related to nuclear receptor activity, ligand-activated transcription factor activity, cytokine receptor binding, steroid hormone receptor activity, DNA-binding transcription factor binding, and RNA polymerase II-specific DNA-binding transcription factor binding. The CC entries were mainly related to membrane microdomain, membrane raft, membrane region, caveolae, and plasma membrane raft.

**Figure 7:**
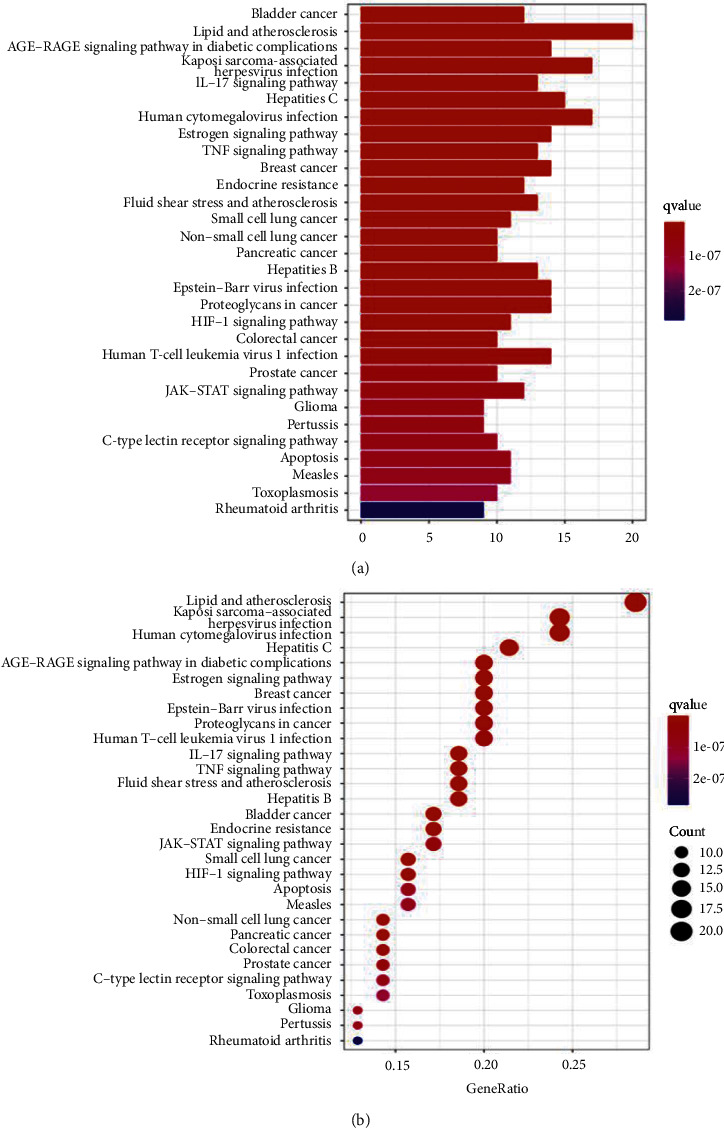
KEGG pathway analysis. (a) Barplot; (b) barble, including BP, MF, and CC. The *y*-axis shows top 30 significantly enriched KEGG pathways, and the *x*-axis displays the number of enrichment genes of these terms (*p* < 0.05). The color of each bar represents the adjusted *p* value, and the length represents the number of enriched genes.

**Figure 8:**
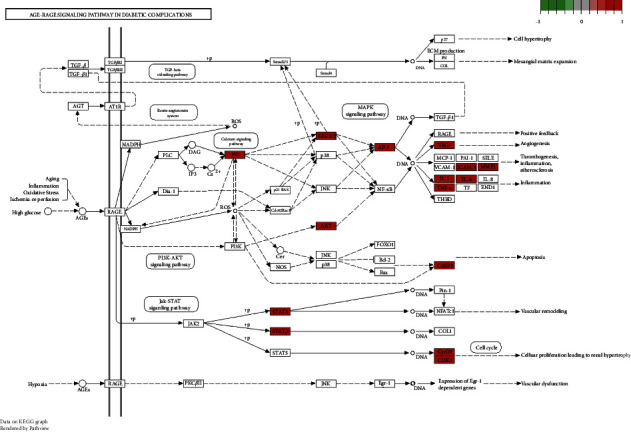
The main nodes of AGE-RAGE signaling pathway. Red nodes mean the genes in the road map present in the SC network in this study.

**Figure 9:**
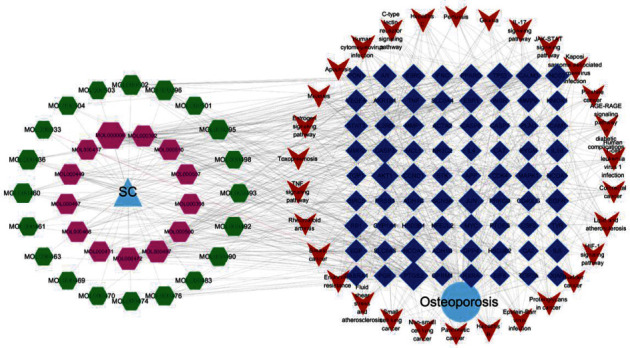
The network of “drug-active ingredients-target genes -pathways -disease.” Yellow triangle represents drug; hexagon node represents the ingredients of SC. Blue square node represents potential treatment targets; red *V* node represents potential OP-related pathways; light blue node represents OP.

Moreover, a total of 139 signaling pathways were yielded through KEGG enrichment analysis, including Bladder cancer, Lipid and atherosclerosis, Kaposi sarcoma-associated herpesvirus infection, AGE-RAGE signaling pathway, IL-17 signaling pathway, Hepatitis C, Human cytomegalovirus infection, Estrogen signaling pathway, Breast cancer, and TNF signaling pathway. As shown in Figure 7, we visualized the top 30 filtered pathways using bubble plot and bar diagram. Among these signaling pathways, AGE-RAGE signaling pathway was chosen to further research (Figure 8); in the picture, red nodes mean the genes in the road map present in the SC network in this study. Finally, a ternary network of “active ingredients—potential targets-signaling pathways” was drawn successfully (Figure 9).

### 3.6. Molecular Docking Results

**Figure 10:**
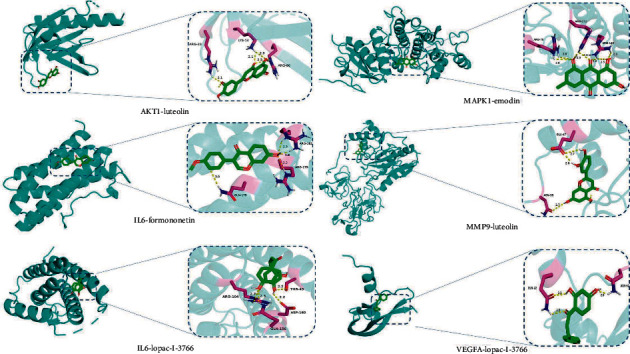
Docking graph between bioactive components and core genes.

10 core genes (MAPK1, VEGFA, MMP9, AKT1, AR, IL6, CALM3, TP53, EGFR, and CAT) were identified using 12 algorithms in CytoHubba. After KEGG enrichment analysis, we searched for OP in the KEGG pathway database and found that there are mainly two pathways directly related to OP and related diseases, including AGE-RAGE signaling pathway and Estrogen signaling pathway. Subsequently, we found that five of the top 10 hub genes (MAPK1, VEGFA, MMP9, AKT1, and IL6) were enriched in AGE-RAGE signaling pathway. Aloe-emodin, emodin, formononetin, licochalcone A, luteolin, and lopac-I-3766 were selected as the top active components for molecular docking with MAPK1, VEGFA, MMP9, AKT1, and IL6 in turn. “The binding energy between drug component ligands and target receptors is an important indicator to evaluate the binding capacity. It is generally considered that the docking affinity is stronger when the binding energy is less than—5.0 kcal/mol, and the docking activity is extremely strong when the binding energy is less than—7.0 kcal/mol.” From the docking results (Tables 3 and 4), it was found that AKT1-luteolin, IL6-formononetin, IL6-lopac-I-3766, MAPK1-emodin, MMP9-luteolin, and VEGFA- lopac-I-3766 had the lower binding energies. It is speculated that SC mainly exerted anti-OP effect through above molecular docking process. Eventually, we chose above protein targets and active component with the lowest docking affinity for visualization (Figure 10).

## 4. Discussion

OP is a metabolic disease caused by various factors, such as age, endocrine, viral infection, and a variety of cytokines. Given that the multitarget and multipathway pharmacological properties of TCM make it more suitable for the treatment of OP, SC is a widely used traditional medicine for promoting blood circulation to remove blood stasis and nourishing blood. Modern pharmacological studies revealed that SC exerted anti-OP through inhibition of osteoclastogenesis and stimulation of chondrogenesis. However, the underlying mechanism remains unclear. The aim is to clarify the potential mechanisms of SC for OP treatment integrated zebrafish model and network pharmacology.

Zebrafish is an ideal animal model in vivo for studying bone deformations for the high similarity of the structure and genetics of bone with human beings [74]. In the present study, we established the zebrafish OP model induced by PNSL to evaluate the effects of SC on the bone formation and examined the bone mineralization by Alizarin red staining, which is a vital dye staining, and the biological mineralization process of zebrafish can be observed directly. Our results indicated that PNSL exposure inhibited osteogenic differentiation and bone mineralization in zebrafish larvae (*p* < 0.05; Figures 1(a) and 1(b)). Compared with DMSO group, treatment with PNSL at 25 *μ*mol/L caused an apparent decrease in the SSA and TOD with values of 51423.33 and 19405.03, respectively, which corresponded to the ventral view of Alizarin Red stained zebrafish skull, as shown in Figure 1(c). After the intervention of SC, TOD and SSA were increased to 295764.70 and 109542.50 at 10 mg/mL, respectively (Figures 1(a) and 1(b)). Besides, SC increased MD by 131.50%, 85.34%, and 69.60%, with partial significance (*p* < 0.001 − 0.01). TF for SC-H, SC-M, and SC-L was 371.43%, 254.54%, and 159.62% (Figures 2(a)–2(c)). Also, the hot plot revealed zebrafish activity degree (Figure 2(d)). These results demonstrated that SC played a positive effect on the function of osteoblastic differentiation and mineralization in zebrafish larvae.

Behavioral changes of zebrafish have been linked to chemical exposure [75, 76]. The behavior analyzer EthoVision XT made it possible to examine numerous motor events and facilitated the quantitative analysis of behavior [77]. The behavioral change of zebrafish is an essential indicator to assess the anti-OP effect of SC. The MD and TF in PNSL group further supported that the construction of osteoporosis model was successful. MD for SC was 60.32%–131.50%, 71.59%–85.34%, and 47.63%–69.60%, respectively. These results with significant differences demonstrated that SC could improve dyskinesia of zebrafish to some extent.

By evaluating the pharmacokinetic characteristics of the chemical ingredients in SC, we identified 33 bioactive components and determined 72 potential therapeutic targets of OP. These common targets of SC were related to the regulation of diverse biological activities, including response to steroid hormone, response to metal ion, cellular response to chemical stress, response to drug, regulation of DNA-binding transcription factor activity, response to oxygen levels, and muscle cell proliferation. These results were consistent with those of the previously reported pathological processes of OP [78–80]. Pathway enrichment analysis indicated that the targets of SC were enriched in diverse pathways implicated in OP pathology, such as AGE-RAGE, lipid and atherosclerosis, IL-17, Estrogen, and TNF signaling pathway.

In this study, 10 core genes (MAPK1, VEGFA, MMP9, AKT1, AR, IL6, CALM3, TP53, EGFR, and CAT) were identified using plug-in CytoHubba in Cytoscape. Subsequently, we found that five of the top 10 hub genes (MAPK1, VEGFA, MMP9, AKT1, and IL6) were enriched in AGE-RAGE signaling pathway. MAPK1, generally known as ERK2, is a member of mitogen-activated protein kinase family and acts as an essential regulator of cell proliferation, differentiation, inflammation, and bone metabolism. It has been demonstrated the activation of MAPK1 could promote osteoblastogenesis and bone mineralization in zebrafish larvae. VEGFA is a signal factor promoting neovascularization and increase vascular permeability. A recent research reported that VEGFA was a latent marker of endothelial dysfunction in postmenopausal osteoporosis. Moreover, through directly targeting VEGFA, MIR-16-5P mitigated the symptom of postmenopausal women with osteoporosis. MMP9 is a member of matrix metalloproteinases family, responsible for extracellular matrix degradation and cleavage of its structural components. A previous study illustrated that MMP9 exerted a crucial role in pathogenesis of osteoporosis, and the inhibitory effect on bone resorption was emerged by inhibiting the expression of MMP9. AKT1, also called Protein Kinase B, is a crucial signal transducer of PI3K/AKT signaling pathway. The phosphorylation of AKT1 modulates the expression of multiple downstream effectors including mTOR‐C1 and FOXO3 proteins and then mitigates OP induced by iron overload. Based on the above analysis results, it is also speculated that SC against OP may play a role through the above process.

GO and KEGG enrichment analysis results showed that the therapeutic targets of SC for diseases mainly enriched in Bladder cancer, Lipid and atherosclerosis, AGE-RAGE signaling pathway, Kaposi sarcoma-associated herpesvirus infection, IL-17 signaling pathway, Hepatitis C, Human cytomegalovirus infection, Estrogen signaling pathway, TNF signaling pathway, and breast cancer. Studies have shown that all of these play a crucial role in the progression of OP. We searched for OP in the KEGG pathway database and found that there are mainly two pathways directly related to OP and related diseases, including AGE-RAGE signaling pathway and Estrogen signaling pathway. Subsequently, we found that five of the top 10 hub genes (MAPK1, VEGFA, MMP9, AKT1, and IL6) were enriched in AGE-RAGE signaling pathway. The AGE-RAGE signaling pathway plays a crucial role in bone remodeling process. In general, AGEs not only induce osteoclastogenesis by upregulation of RANKL mRNA, but they also affect osteoblasts by suppressing cell growth, promoting apoptosis, and downregulating differentiation, which impairs mineralization. It was reported that AGEs upregulated the expression of RAGE in human MSCs and then AGEs interacted with RAGE and increased the expression of TGF-b mRNA, which suppressed bone mineralization and destroyed bone remodeling [33]. In this study, five of the top 10 hub genes were enriched in the AGE-RAGE signaling pathway, so we speculated that the underlying mechanism of SC on OP might be mainly the inhibition of AGE-RAGE signaling pathway.

## 5. Conclusion

Compared with model group, SC significantly increased the SSA and TOD at 10 mg/mL and improved the locomotor activity in a dose-dependent manner (*p* < 0.001). 33 components of SC were associated with 72 OP-related genes including 10 core genes. AGE-RAGE signaling pathway was screened out as the principal pathway of SC in anti-OP. The bioactive components (Aloe-emodin, Emodin, Formononetin, Licochalcone A, Luteolin, and Lopac-I-3766) have excellent affinity to core genes (MAPK1, VEGFA, MMP9, AKT1, and IL6). *Conclusion.* SC had the hierarchical network characteristics of “multicomponents/multitargets/multifunctions/multipathways” in reversing OP, but AGE-RAGE signaling pathway may be the main regulatory mechanism. We have demonstrated the anti-OP effect of SC and revealed its underlying mechanism by adopting zebrafish model and network pharmacology. SC mainly regulated AGE-RAGE signaling pathway to exert anti-OP therapeutic effect.

## Figures and Tables

**Table 1:** All bioactive components of Spatholobi Caulis.

Molecule ID	Component	Molecular weight	Oral bioavailability	Drug like
MOL000295	Alexandrin	576.95	20.63	0.63
MOL000296	Hederagenin	414.79	36.91	0.75
MOL000033	(24S)-24-Propylcholesta-5-ene-3beta-ol	428.82	36.23	0.78
MOL000358	Beta-sitosterol	414.79	36.91	0.75
MOL000392	Formononetin	268.28	69.67	0.21
MOL000417	Calycosin	284.28	47.75	0.24
MOL000436	lopac-I-3766	256.27	87.51	0.15
MOL000449	Stigmasterol	412.77	43.83	0.76
MOL000460	(Z)-1-(2, 4-Dihydroxyphenyl)-3-(3, 4-dihydroxyphenyl) prop-2-en-1-one	272.27	83.78	0.17
MOL000461	3,7-Dihydroxy-6-methoxy-dihydroflavonol	302.3	43.8	0.26
MOL000463	16844-71-6	428.82	27.34	0.76
MOL000467	Castanin	298.31	23.54	0.27
MOL000468	8-o-Methylreyusi	298.31	70.32	0.27
MOL000469	3-Hydroxystigmast-5-en-7-one	428.77	40.93	0.78
MOL000470	8-C-*α*-L-Arabinosylluteolin	418.38	35.54	0.66
MOL000471	Aloe-emodin	270.25	83.38	0.24
MOL000472	Emodin	270.25	24.4	0.24
MOL000474	(-)-Epoxycaryophyllene	220.39	35.94	0.13
MOL000476	Physcion	284.28	22.29	0.27
MOL000483	(Z)-3-(4-Hydroxy-3-methoxy-phenyl)-N-[2-(4hydroxyphenyl)ethyl]acrylamide	313.38	118.35	0.26
MOL000490	Petunidin	317.29	30.05	0.31
MOL000492	(+)-Catechin	290.29	54.83	0.24
MOL000493	Campesterol	400.76	37.58	0.71
MOL000497	Licochalcone a	338.43	40.79	0.29
MOL000498	Isoorientin	448.41	23.3	0.76
MOL000500	Vestitol	272.32	74.66	0.21
MOL000501	Consume close grain	302.3	68.12	0.27
MOL000502	Cajinin	300.28	68.8	0.27
MOL000503	Medicagol	296.24	57.49	0.6
MOL000504	Kadsurin	456.58	25.22	0.84
MOL000507	Psi-baptigenin	282.26	70.12	0.31
MOL000510	Olmelin	284.28	25.21	0.24
MOL000006	Luteolin	286.25	36.16	0.25

**Table 2:** Overlapping targets of drug potential targets and disease-related targets.

Target name	Symbol
RAC-alpha serine/threonine-protein kinase	AKT1
Cellular tumor antigen p53	TP53
Interleukin-6	IL6
Transcription factor AP-1	JUN
Mitogen-activated protein kinase 1	MAPK1
Vascular endothelial growth factor A	VEGFA
Tumor necrosis factor	TNF
Myc proto-oncogene protein	MYC
Signal transducer and activator of transcription 3	STAT3
Epidermal growth factor receptor	EGFR
Caspase-3	CASP3
Pro-epidermal growth factor	EGF
Matrix metalloproteinase-9	MMP9
Prostaglandin G/H synthase 2	PTGS2
Estrogen receptor	ESR1
G1/S-specific cyclin-D1	CCND1
Caspase-8	CASP8
Peroxisome proliferator activated receptor gamma	PPARG
Interleukin-1 beta	IL1B
Catalase	CAT
72 kDa type IV collagenase	MMP2
Interleukin-4	IL4
Androgen receptor	AR
Intercellular adhesion molecule 1	ICAM1
Granulocyte-macrophage colony-stimulating factor	CSF2
Heme oxygenase 1	HMOX1
Interferon gamma	IFNG
Progesterone receptor	PGR
Cell division protein kinase 4	CDK4
Interstitial collagenase	MMP1
Induced myeloid leukemia cell differentiation protein Mcl-1	MCL1
Nitric oxide synthase, inducible	NOS2
Urokinase-type plasminogen activator	PLAU
Amyloid beta A4 protein	APP
Estrogen receptor beta	ESR2
Glutathione S-transferase P	XIAP
CD40 ligand	CD40LG
Retinoblastoma-associated protein	RB1
Nuclear factor erythroid 2-related factor 2	NFE2L2
Cytochrome P450 1A1	CYP1A1
Caspase-7	CASP7
Nuclear receptor coactivator 2	NCOA2
Nuclear receptor coactivator 1	NCOA1
Baculoviral IAP repeat-containing protein 5	BIRC5
DNA topoisomerase 2-alpha	TOP2A
Mu-type opioid receptor	OPRM1
Protein kinase C delta type	PRKCD
Tyrosine-protein kinase BTK	BTK
DNA topoisomerase 1	TOP1
Retinoic acid receptor RXR-alpha	RXRA
Calmodulin	CALM3
Aldose reductase	AKR1B1
Interleukin-2	IL2RB
Dipeptidyl peptidase IV	DPP4
Actin, aortic smooth muscle	ACTA2
Insulin receptor	INSR
Prostaglandin E synthase	PTGES
Sodium-dependent noradrenaline transporter	SLC6A4
Serum paraoxonase/arylesterase 1	PON1
3 Beta-hydroxysteroid dehydrogenase/Delta 5-->4-isomerase type 1	HSD3B1
Tyrosinase	TYR
Mineralocorticoid receptor	NR3C2
3 beta-hydroxysteroid dehydrogenase/Delta 5-->4-isomerase type 2	HSD3B2
Trypsin-3	PRSS3
Alcohol dehydrogenase 1B	ADH1B
Alcohol dehydrogenase 1C	ADH1C
Sodium-dependent dopamine transporter	SLC6A3
Carbonic anhydrase II	CA2
Glycogen phosphorylase, muscle form	PYGM
Gamma-aminobutyric acid receptor subunit alpha-1	GABRA1
Sodium channel protein type 5 subunit alpha	SCN5A
Eukaryotic translation initiation factor 6	EIF6

**Table 3:** Docking energy and interacting residues between receptors and ligands.

Receptor	Ligand	Affinity (kcal/mol)	Residues
AKT1	Aloe-emodin	−6.6	LEU-52, GLN47, GLU40
	Emodin	−6.8	TRP-11, HIS-13, GLU-91
	Formononetin	−7	TRP-11, HIS-13, GLU-91
	Licochalcone a	−6	TRP-11, HIS-13, GLU-95
	Luteolin	−8.4	ARG-23, LYS-14, ARG-86
	lopac-I-3766	−6.3	LYS-8, GLU-95, HIS-89

IL6	Aloe-emodin	−6.9	ASN-63, THR-143
	Emodin	−6.9	ASN-144, THR-143, LEU-147
	Formononetin	−8.8	GLN-175, ARG-182, ARG-179
	Licochalcone a	−6.6	ARG-182, GLN-175
	Luteolin	−7.1	ASP-34, ARG-179
	lopac-I-3766	−8.6	ARG-104, GLN-156, ASP-160, THR-43

MAPK1	Aloe-emodin	−7.8	ASP-111, GLU-71, LYS-54
	Emodin	−9.1	ARG-70, ARG-172, ARG-148
	Formononetin	−8.1	ASN-154, LYS-54
	Licochalcone a	−7.3	ARG-67
	Luteolin	−8.6	LYS-114, LYS-54
	lopac-I-3766	−7.4	GLN-105, LEU-156

MMP9	Aloe-emodin	−7.6	GLU-47, ARG-51
	Emodin	−7.8	ASN-38, ARG-95, ARG-51
	Formononetin	−7.8	THR-426, LEU-188
	Licochalcone a	−6.5	ARG-143
	Luteolin	−8	GLU-47, ASN-38
	lopac-I-3766	−7.3	HIS-401, THR-426

VEGFA	Aloe-emodin	−6.3	ASP-34
	Emodin	−6.5	ASP-34, ILE-46
	Formononetin	−5.9	SER-50
	Licochalcone a	−5.8	GLN-37
	Luteolin	−6.2	ASP-34
	lopac-I-3766	−7.7	GLN-22, ASN-62

**Table 4:** Bonding position and H-bond interaction between receptors and ligands.

Receptor	Ligand	Position	Receptor	Interaction	Distance
AKT1	Aloe-emodin	O (5)	GLN-47	H-donor	2.5
		O (3)	GLU-40	H-acceptor	3.4
		O (1)	LEU-52	H-donor	2.6
	Emodin	O (5)	GLU-91	H-donor	3.3
		O (3)	HIS-13	H-acceptor	3.4
		6-ring	TRP-11	H-acceptor	3.7
	Formononetin	6-ring	TRP-11	H-acceptor	3.5
		O (4)	GLU-91	H-donor	3
		O (2)	HIS-13	H-acceptor	3
	Licochalcone a	O (1)	GLU-95	H-donor	3.1
		O (2)	HIS-13	H-acceptor	3.2
		O (3)	TRP-11	Pi-H	3.8
	luteolin	O (6)	ARG-23	H-acceptor	2.1
		O (4)	LYS-14	H-acceptor	2.1
		O (4)	ARG-86	H-acceptor	2.5
		O (2)	LYS-14	H-acceptor	2.3
	lopac-I-3766	O (4)	LYS-8	H-acceptor	2
		6-ring	GLU-95	Pi-H	3.5
		O (1)	HIS-89	H-donor	3.4
IL6	Aloe-emodin	O (1)	ASN-63	H-donor	3.3
		O (3)	THR-143	H-acceptor	3.3
		O (5)	THR-143	H-acceptor	3.6
	Emodin	O (3)	ASN-144	H-acceptor	3.1
		O (3)	THR-143	H-acceptor	3.3
	Formononetin	6-ring	GLN-175	H-acceptor	3
		O (1)	ARG-182	H-acceptor	2.3
		O (1)	ARG-182	H-acceptor	1.8
		O (1)	ARG-179	H-acceptor	2.2
	Licochalcone a	O (1)	ARG-182	H-acceptor	2.5
		6-ring	GLN-175	H-donor	2.2
	Luteolin	O (5)	ASP-34	H-donor	3.3
		O (1)	ARG-179	H-acceptor	2.8
		O (1)	ARG-179	H-acceptor	2.7
	lopac-I-3766	O (4)	ARG-104	H-acceptor	2.3
		O (4)	ARG-104	H-acceptor	2.7
		O (1)	THR-43	H-acceptor	2.2
		O (1)	ASP-160	H-donor	2.2

MAPK1	Aloe-emodin	O (4)	GLU-71	H-donor	3.6
		O (4)	LYS-54	H-acceptor	2.2
		6-ring	ASP-111	Pi-H	3.3
	Emodin	O (1)	ARG-70	H-acceptor	2.8
		O (2)	ARG-172	H-acceptor	2.3
		O (2)	ARG-148	H-acceptor	2
	Formononetin	O (4)	ASN-154	H-donor	2.6
		O (2)	LYS-54	H-acceptor	2
	Licochalcone a	O (1)	ARG-67	H-acceptor	1.8
		O (1)	ARG-67	H-acceptor	3
	Luteolin	O (6)	LYS-114	H-acceptor	2.6
		O (2)	LYS-54	H-donor	2.3
	lopac-I-3766	O (1)	GLN-105	H-donor	3.7
		O (2)	LEU-156	H-acceptor	3.6

MMP9	Aloe-emodin	O (5)	GLU-47	H-donor	2.6
		O (5)	ARG-51	H-acceptor	2.2
		O (5)	ARG-51	H-acceptor	3.4
	Emodin	O (5)	ASN-38	H-donor	3.6
		6-ring	ARG-51	H-acceptor	2.7
	Formononetin	O (1)	THR-426	H-acceptor	2.5
	Licochalcone a	O (2)	ARG-143	H-acceptor	2.1
		O (2)	ARG-143	H-acceptor	2.6
	Luteolin	O (1)	ASN-38	H-donor	2.7
		O (5)	GLU-47	H-donor	2
		O (6)	GLU-47	H-donor	2.7
	lopac-I-3766	O (4)	THR-426	H-acceptor	2.6

VEGFA	Aloe-emodin	O (5)	ASP-34	H-donor	2.1
		O (5)	ASP-34	H-donor	2.6
	Emodin	O (4)	ASP-34	H-donor	3.3
		O (5)	ILE-46	H-donor	3.6
	Formononetin	O (1)	SER-50	H-acceptor	3.6
		O (1)	SER-50	H-donor	2.4
	Licochalcone a	O (1)	GLN-37	H-donor	2.6
	Luteolin	O (5)	ASP-34	H-donor	2.1
		O (5)	ASP-34	H-donor	2.2
	lopac-I-3766	O (2)	GLN-22	H-acceptor	2.5
		O (3)	GLN-22	H-donor	2.3
		O (1)	ASN-62	H-acceptor	2

## Data Availability

The original contributions presented in the study are included in the article andsupplementary materials. Further inquiries can be directed to the corresponding authors.
